# Does the cowl make the monk? The effect of military and Red Cross uniforms on empathy for pain, sense of agency and moral behaviors

**DOI:** 10.3389/fpsyg.2023.1255835

**Published:** 2023-10-03

**Authors:** Guillaume P. Pech, Emilie A. Caspar

**Affiliations:** ^1^Consciousness, Cognition and Computation lab, Center for Research in Cognition and Neuroscience, Université Libre de Bruxelles, Brussels, Belgium; ^2^Moral & Social Brain lab, Department of Experimental Psychology, Ghent University, Ghent, Belgium

**Keywords:** enclothed cognition, sense of agency, empathy for pain, prosocial behavior, coercion

## Abstract

According to the embodied cognition framework, cognitive functions are not confined to the brain but are also shaped by the mutual interactions between the brain, body, and external environment. In this regard, a theory developed in 2012, called enclothed cognition, suggests an effect on wearing specific clothing on various psychological processes. However, the neuro-cognitive mechanisms underlying the impact of clothing on behavior have received less systematic investigation. The present study examined the influence of clothing on prosocial behaviors, and focused on sense of agency, and empathy for pain as neuro-cognitive processes of interest. Participants (40 in total) wore civilian, military, and Red Cross uniforms. They were paired up and assigned as either agents or victims. Agents had the option to administer real electric shocks to victims for a monetary reward of +€0.05. They could choose to shock freely (free condition) or follow the experimenter’s instructions (coerced condition). We measured prosocial behavior by counting the number of shocks prevented, neural empathic response using electroencephalography with the P3 and the LPP, and sense of agency through an implicit method based on interval estimates. Findings showed that wearing the Red Cross uniform led to more prosocial behavior compared to civilian clothing. The Red Cross uniform also increased neural response to pain when participants witnessed shocks, compared to civilian or military clothing. Moreover, wearing a military uniform increased the sense of agency in the free condition, as compared to civilian clothing. This study broadens our knowledge on the impact of enclothed cognition on cognitive and psychological processes.

## Introduction

Can the uniforms we wear influence our behavior? In traditional cognitive science, the focus has been on cognition as a process confined to the brain (Gallagher, 2017). However, a different perspective called embodied cognition suggests that our cognitive processes are shaped by interactions among the brain, body, and environment (Varela et al., 1991). This viewpoint recognizes that perception plays a role in shaping cognition. Numerous studies integrating the concept of embodied cognition have provided examples of how various factors can influence cognition. For instance, facial muscle activations associated with emotions can amplify the intensity of those emotions (Coles et al., 2019), and hand grasping actions can be influenced by the presentation of congruent or incongruent pictures (Till et al., 2014). Another intriguing aspect, which has been less extensively studied, is the influence of the symbolic meaning of the clothes we wear on our cognition and behavior. In Adam and Galinsky (2012) found that wearing a doctor uniform improved performance in a Stroop task compared to wearing a painter uniform. They referred to this approach as *enclothed cognition* within the framework of embodied cognition. Additional studies have examined the effects of specific clothing on various psychological processes (Adam and Galinsky, 2019). For example, past research has shown that wearing certain clothes can influence mental abstraction (Burger and Bless, 2017), problem-solving (Van Stockum and DeCaro, 2014), and prosocial behavior (López-Pérez et al., 2016). However, the neuro-cognitive mechanisms underlying the impact of clothing on behavior have received less systematic investigation. In the present study, our aim was to examine how the uniforms we wear influence prosocial behaviors. Prosocial behaviors refer to actions intended to benefit others (Eisenberg and Miller, 1987; Pfattheicher et al., 2022). These behaviors are considered significant indicators of social and economic development in almost all societies (Meier, 2006; Andriani and Sabatini, 2015), and as a result, societies strive to promote prosociality due to its potential benefits (Baumsteiger, 2019). While measuring prosociality often involves using fictitious scenarios or self-reported questionnaires (Greene et al., 2001; Agerström and Björklund, 2009; Pavey et al., 2011; Baumsteiger and Siegel, 2019), such methods have limited ecological validity. Consequently, other studies have adopted more ecologically valid approaches by examining real behaviors with tangible consequences for others (Darley and Latane, 1968; Murphy and Ackermann, 2013; Van Lange et al., 2013). For example, costly helping tasks assess prosocial behaviors by examining participants’ decisions to refuse personal monetary gains in order to prevent another person from experiencing a painful shock (Hein et al., 2010; Caspar et al., 2016).

In the literature, several neuro-cognitive processes have been linked to prosocial behaviors (Baumsteiger, 2019; Laguna et al., 2020) and in the present study we have decided to focus on empathy for pain and the sense of agency (Stocks et al., 2009; Baumsteiger, 2019; Caspar et al., 2022). Empathy for pain has been defined as our capacity to feel the pain of others (Singer et al., 2004). To measure empathy, explicit methods such as self-reported questionnaires can be used, but they are sensitive to self-reported bias such as social desirability (Neumann and Westbury, 2011; Rosenman et al., 2011; Althubaiti, 2016; Sassenrath, 2020). Another way to measure empathy is to use neuroimaging methods, as an extensive literature has indeed shown that seeing another individual in pain triggers an empathic response in the brain of the observer, termed as the ‘pain matrix’ (Decety, 2011; Bernhardt and Singer, 2012; Keysers and Gazzola, 2014; Lamm et al., 2019), notably in the anterior insula, in the dorsal anterior cingulate cortex, and the cerebellum (Singer et al., 2004). In electroencephalography studies, a recent meta-analysis suggested that the P3 and the late positive potential (LPP) recorded over centro-parietal area are robust event-related potentials (ERP) to measure the neural empathic response to the pain of others (Coll, 2018).

The sense of agency refers to the subjective experience of being the author of our actions and consequently being responsible for their outcomes (Gallagher, 2000). In the literature, explicit methods have been employed to measure the sense of agency. However, similar to self-reported measures for empathy, these methods can be influenced by social desirability biases (Yoshie and Haggard, 2013). As an alternative, researchers have used an implicit method based on time perception (Haggard, 2017). In traditional time perception tasks, participants are asked to estimate the duration of a time interval between an action (e.g., pressing a button) and its resulting consequences (e.g., hearing a beep). Findings consistently reveal that participants tend to perceive time intervals as shorter when they have performed the action voluntarily compared to when the action was performed involuntarily (Moore and Haggard, 2010). This phenomenon is known as temporal binding. Temporal binding appears as a consistent phenomenon that emerges when there is a shift in the sense of agency (Haggard, 2017; Hoerl et al., 2020; Wen and Imamizu, 2022), leading to a compression of perceived time when individuals have a stronger sense of agency over their actions (for a meta-analysis, see Tanaka et al., 2019). It is crucial to bear in mind that this effect does not directly measure the sense of agency itself; rather, it serves as a proxy for it. Intriguingly, initially, temporal binding was believed to primarily reflect changes in intentionality (Haggard, 2017). Nevertheless, evidence points towards the possibility that this temporal compression might also stem from an augmented perception of causality (Hoerl et al., 2020).

Although empathy for pain and the sense of agency have been predominantly studied separately in the fields of social and cognitive neuroscience, several studies have demonstrated a positive relationship between them, indicating that they mutually influence each other (Koban et al., 2013; Lepron et al., 2015; Caspar et al., 2022). Moreover, both empathy for pain and the sense of agency have been found to play crucial roles in explaining prosocial behaviors (Bandura et al., 2003; Hein and Singer, 2010; Reniers et al., 2012; Lamm and Majdandžić, 2015; Gallo et al., 2018; Waller et al., 2020; Caspar et al., 2020a). Given the importance of empathy for pain and the sense of agency in understanding prosocial behaviors, they emerge as reliable factors to consider when examining the influence of uniforms on such behaviors.

Previous research conducted by López-Pérez et al. (2016) explored the impact of wearing specific clothes on empathy and prosocial behaviors. Participants in their study were instructed to wear either a nurse uniform or a cleaner uniform. The findings revealed that individuals wearing the nurse uniform demonstrated higher levels of prosocial behavior in the Zurich Prosocial Game (Leiberg et al., 2011) and exhibited increased scores on the Empathic Response Scale (Batson et al., 1987) compared to those wearing the cleaner uniform. This study suggests that both prosocial behavior and empathy can be influenced by the uniform one wears. However, given that prosocial behavior encompasses a wide range of behaviors, it would be beneficial to replicate these findings in different contexts. Furthermore, the reliance on self-reported measures of empathy in the study raises the possibility of social desirability bias. To extend these findings, our study aims to employ a neurophysiological measurement of empathy, thereby avoiding potential biases associated with self-report measures. To the best of our knowledge, no previous studies have investigated the influence of uniforms on the sense of agency. However, a prior study by Caspar et al. (2020b) compared military individuals wearing their military outfits to civilians wearing their civilian outfits. Using the method of interval estimates as an implicit marker of the sense of agency, the results indicated that military cadets and privates had a diminished sense of agency when they had the freedom to decide their actions compared to civilians. The authors concluded that the highly hierarchical military environment had a negative impact on the sense of agency. Notably, in this study, a confounding factor was present as participants wore different outfits corresponding to their respective roles, raising the question of whether the diminished agency was solely due to the military environment or if the act of wearing a military uniform itself played a role.

In this study, we aimed to use the paradigm of Caspar et al. (2020b), which allows for exploring the effects of wearing specific outfits and has successfully measured prosocial behaviors (Caspar et al., 2017), the sense of agency (Caspar et al., 2016), and the neural empathic response to others’ pain (Caspar et al., 2020a) within a single relatively ecological task. Two participants were assigned the roles of agent and ‘victim.’ Agents had the option to freely decide or receive instructions from the experimenter to administer real electric shocks to the ‘victim’ in exchange for a small monetary gain. Prosocial behaviors were assessed based on the agent’s decision to renounce increasing their remuneration in order to prevent the ‘victim’ from receiving a shock in the free condition. The neural empathic response was measured by analyzing the amplitude of the P3 and the LPP when participants observed the shocks being delivered to the ‘victim.’ The sense of agency was evaluated using the method of interval estimates. To modulate prosocial behavior, empathy (based on López-Pérez et al., 2016), and agency (following Caspar et al., 2020b), three different outfits were employed in a within-subject design: a Red Cross uniform representing healthcare, a military uniform, and participants’ own civilian outfits as a control. Based on the findings of López-Pérez et al. (2016), we hypothesized that wearing the Red Cross uniform would result in higher prosociality in the free condition (i.e., fewer shocks delivered) and a greater neural response to the ‘victim’s’ pain compared to the military uniform or civilian outfit, regardless of the experimental condition. Drawing from Caspar et al.’s (2020b) results, we expected a decrease in the sense of agency when participants wore the military uniform compared to their civilian outfit or the Red Cross uniform. Given that the testing occurred over three consecutive days, a decline in empathy might be anticipated, as previous studies have shown reduced empathy among healthcare staff over time (Decety et al., 2010; Neumann et al., 2011).

## Methods

### Participants

We recruited forty participants(22 males, 18 females) by dyads without selection for preference for dominance handing. We did not mix gender in dyads, so we had male–male or female–female dyads. We calculate the sample size for repeated measures ANOVA with a 2*3 within design including condition (i.e., free and coerced) and uniforms (i.e., Red Cross, military, civilian) on G-Power (Faul et al., 2007). As no previous studies used a similar experimental approach, we used a small-to-medium effect size *f* of 0.175 to calculate the sample size. (Faul et al., 2007). We entered one group with the 2*3 (=6) number of measurements. The correction among repeated measures of was set to 0.5 and the nonsphericity correction to 1. To achieve a power of 0.80 for this effect size, the estimated sample size was 37. We increased the sample size up to 40 to compensate for loss of data. We recruited diverse participants through advertisements on social medias and on job recruitment websites. Inclusion criteria included being between 18 and 36 years old, having a corrected or normal vision, not having participated in a similar experiment before, not having an history of neurological or psychiatric disorder, not being a military or a first aid for the Red Cross, having a haircut that fits with electroencephalography (EEG) recordings (i.e., no dreadlocks, not bold) and not applying at the same time as a friend or a relative. Exclusion criteria were determined prior the data acquisition. They included: (1) failure to discriminate between time intervals, (2) bad signal-to-noise ratio in EEG, (3) not paying attention to the task, and (4) full disobedience in the Coerced condition. For time intervals (see exclusion criteria 1), to identify participants for whom the action-tone intervals did not gradually increase with action-tone intervals, we performed a linear trend analysis (LTA) with contrast coefficients −1, 0, 1 for the three delays we used, similarly to previous studies (Hess et al., 2001; Caspar et al., 2020b). This significant trend is a way to ensure that participants reported higher estimation when the intervals were higher. For example, when the interval was 200 ms, participants were expected to report a lower estimation than when the interval was 500 ms. Thus, a linear trend should appear between 200, 500 and 800 ms. If no significant linear trend was observed, we considered that the participant did not pay attention to the interval durations. The data of 8/40 participants were excluded due to a non-significant LTA. Other data were kept for those participants. For example, participants removed due to the time estimation discrimnation where not removed from the EEG, prosocial behavior nor questionnaires measurements. Regarding EEG recordings, we had to exclude 2/40 participants due to a bad signal-to-noise ratio (i.e., bad signal on the electrodes of interest or noisy reference electrodes). Further, 13/40 participants did not send enough shocks (<5) to have reliable measurements in the Free condition. Some participants sent enough shocks on some days, but not sufficiently for reliable analyses on other days, thus preventing to use their data in our within-subject design (4/13). These 13 participants have thus not been analyzed for the EEG and interval estimates and have been excluded for these measurements as relying on an averaging of several values. However, these 13 participants had been kept for prosocial behaviors and for the questionnaires as relying on a single value. We finally had *N* = 40 participants for the analyses on prosocial behavior and the questionnaires, *n* = 25 participants for the EEG measurement and *n* = 23 participants for the interval estimates measurement some of the participants disobeyed to some trials but none of them disobeyed to all the instructions in the coerced condition. All participants were paid 30 euros for their participation and were instructed that they could increase their own monetary gain based on their decisions during the task. The mean age of participants was 22.65 (SD = 3.66). The study was approved by the local ethical committee of the university (Ref: P2019/484) and respected the Declaration of Helsinki.

### Procedure and material

**Figure 1:**
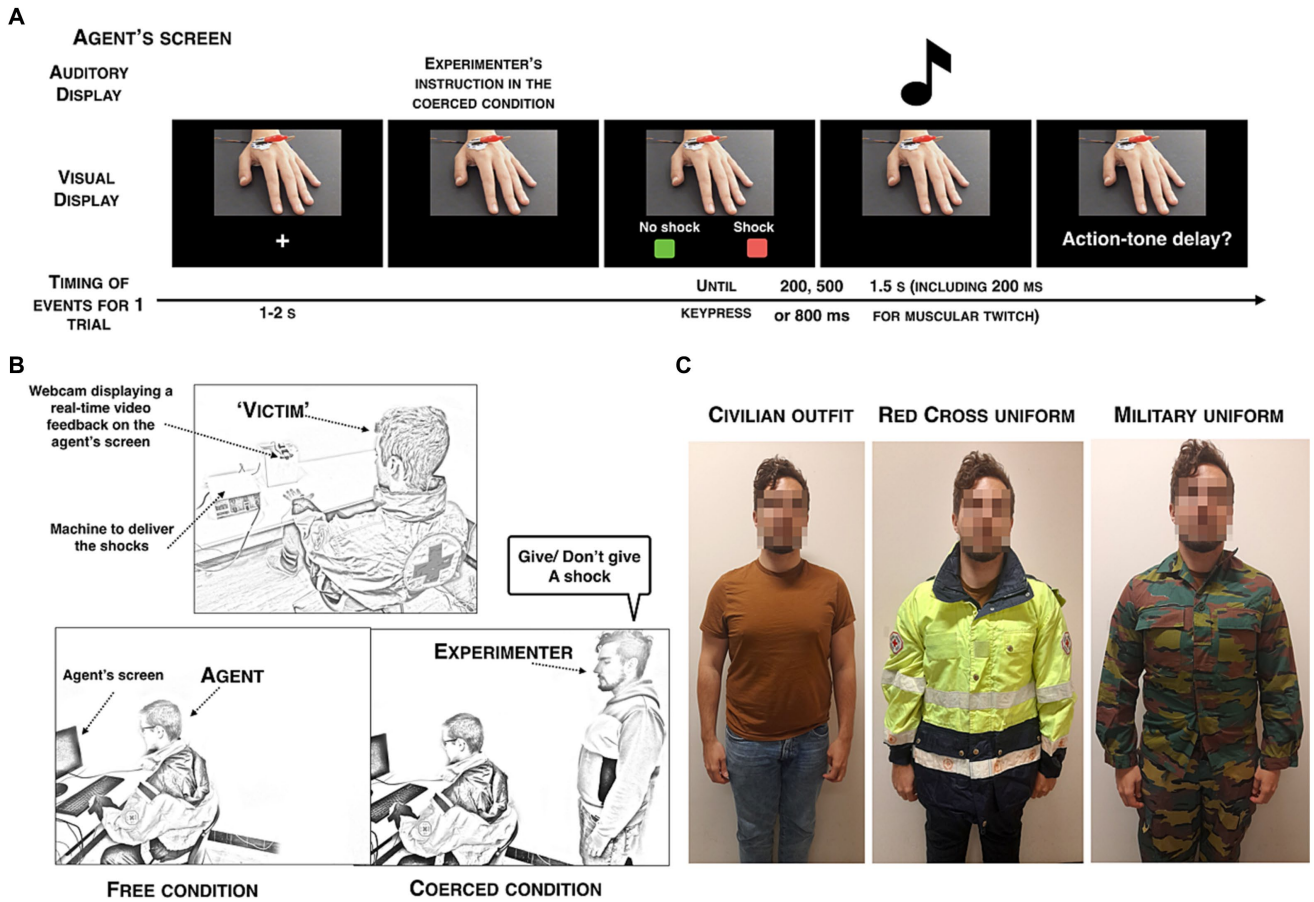
**(A)** Visual display of the agent’s screen. **(B)** Visual display of the experimental procedure. Agent and ‘victim’ were located in different rooms. The victim’s left hand was connected to two electrodes and placed in front of a camera. The agent was either free to decide (free condition) or coercively instructed (coerced condition) to deliver or not a shock to the victim in exchange for +€0.05. **(C)** Visual display of the three different clothing used during the experiment.

Detailed information about the experiment was provided in the advertisement for the task, ensuring that participants were aware of all the aspects of the study before applying. All the dyads came on three consecutive days. Each day, they were wearing a different uniform: Red Cross uniform, military uniform, or their own civilian outfit (see Figure 1C). The first day was slightly different. First, we re-explained the task in details and asked participants if they had any questions. Then, informed consents were signed by the two co-participants, simultaneously, ensuring that they were each aware of the other’s consent. They were reminded their right to withdraw from the study at any time, without any financial loss. Finally, they were provided a training to discriminate intervals in the time interval estimation task for 10 trials.

The experimental procedure was similar across the three testing days in a within-subject design. When participants came to the lab, we designated randomly one out of two rooms and told them that they could drop their personal belongings in that room. Either the military uniform or the Red Cross uniform was already placed in that room, so they only discovered the clothing they had to wear on the testing day, without knowing which uniform they would wear on the next day. In the case of the civilian clothing condition, participants wore their own clothing during the experimental session. The two participants wore the same clothing on the same testing day. On each day, they were asked to watch themselves in the mirror that was located in their room in order to ensure that the uniform was correctly put on. This procedure also ensured that participants would take the time to process the uniform they were currently wearing and to see themselves wearing it. Order of the clothing conditions was counterbalanced between participants and days.

Participants were randomly assigned to either the role of the agent or the role of the ‘victim’, but they were offered the possibility to decide the role they wanted to start with. These roles were reversed mid-way through the experiment, making the procedure fully reciprocal, similarly to the method used by Caspar et al. (2020b). The agent and the ‘victim’ were seated in two different rooms, thus not facing each other, due to Covid safety procedures. The agent wore a 32-electrode cap to record his brain activity. There were two experimental conditions (i.e., free and coerced) composed each of 60 trials. Order of the conditions was counterbalanced between participants. In the coerced condition, participants received instructions from the experimenter, located behind the agent, to send or not a real mildly painful electric shock to the ‘victim’ on each trial. The experimenter ordered randomly the agent to send a shock on 30/60 trials and not to send a shock on the remaining 30/60 trials (see Figure 1B). Participants were told, in the case they asked or tried to disobey, that for the sake of the experiment it was better to follow the instructions but that we could not force them to follow our instructions for ethical reasons. If they continued to disobey, we did not tell them anything more and let them disobey. In the free condition, they could freely choose between delivering a shock or not during 60 trials. We explained to the participants that this decision was their own and confirmed that they were totally free. Each shock sent to the ‘victim, be it in the free or in the coerced condition, was rewarded by +€0.05.

Real painful shocks were used for three main reasons. First, to trigger an empathic reaction in the brain of the observer, pain must be perceived. Second, unlike the studies of Milgram, the present paradigm relies on the non-use of deception, which can alter participants’ trust in the experimenter. Participants here were thus entirely aware of the entire procedure, and we had no hidden information, except the main hypotheses and predictions of the present study. Third, by using real pain, we were able to capture more ecological behaviors as our participants knew that all their decisions had real consequences.

At the beginning of the experiment, we determined participants’ own pain threshold with the electric shock device (i.e., Digitimer DS7A). We first determined the pain threshold for the participant starting as agent, and at the same time, we trained the participant starting as ‘victim’ to perform the time interval estimation task. To detect the pain threshold, we connected two electrodes to the left hand of the participant on the muscle between the index and the thumb in order to produce a visible muscle twitch. We increased the intensity by step of 1 mA starting from 0 until the detection threshold, and by step of 2 mA from the detection threshold until the pain threshold, as Caspar et al. (2016, 2020b) did. Participants were told to indicate when the shock was painful. When they mentioned that the shock was painful, we asked confirmation by asking them if the shock was painful or rather unpleasant and if we could continue to increase. We ensured that participants knew that their selected threshold would not decrease or increase during the experiment, and that they selected a pain threshold that was nonetheless tolerable. They were told that before starting the role of the ‘victim’, they could still ask us to change the threshold. When the experiment started, ‘victims’ were asked to place their left hand on a table positioned in the field of view of a camera and asked not to move their hand during the entire experimental session. The ‘victim’ was invited to watch a neutral documentary to make the time pass.

Each trial began with a fixation cross of 2 s (see Figure 1A). Then, participants saw two rectangles displayed on the bottom of the screen, a red one labelled ‘SHOCK’ and a green one labelled ‘NO SHOCK’. To avoid motor habituation, the two rectangles were randomly located either on the left or on the right. Agents freely chose or received the instruction to press one of the two buttons, which was followed by a tone (400 Hz, 200 ms) played 200, 500 or 800 ms after the keypress. Agents were asked to estimate the elapsed time between their own keypress and the beep onset. They were told to report a number between 0 and 1,000 ms on a paper sheet located in front of them on each trial. They were told to provide precise numbers and to avoid rounding. If a shock was sent to the ‘victim’, the shock was delivered at the exact same time as the tone to avoid temporal bias. Participants were instructed to wait until appearance of the question about the action-tone delay on the screen before answering on the paper sheet. We also asked them to carefully look at the victim’s hand between the keypress and the question so to avoid producing noise in the EEG signals.

All the participants had 60 trials in two experimental conditions (free and coerced) on three consecutive testing days, which resulted in a total of 360 trials. At the end of each experimental session, participants were asked to report what they felt regarding the outfit they wore during the session. We asked them three questions [(1) “*How much do I feel that this outfit had an effect on my behaviors?*” – (2) “*How much do I feel that my state of mind was influenced by this outfit?” –* (3) *“To what extent I behave as a military/first aid worker/civilian during the experimental session?”*]. Participants had to answer on a Likert-type scale ranging from 0 (not at all) to 6 (very much) for the first two questions and ranging from 1 (totally disagree) to 7 (entirely agree) for the third question.

### EEG recordings

We used a Biosemi 32-electrode electroencephalography (EEG) system with four external electrodes. Two of the external electrodes were placed on the left and right mastoids and the two others external electrodes were placed to control for horizontal eye movements. All the data were recorded with the Actiview software. Amplified voltages were sampled at 2,048 Hz. To filter and clean the data, we used the Matlab r2018a software and the Fieldtrip toolbox (Oostenveld et al., 2011). We applied a bandpass filter between 0.1 Hz and 30 Hz. To reference the signal, we subtracted the average of all the electrodes to each electrode instead of the mastoids plugged. Unfortunately, the speakers we used introduced a noise in each electrode, including the mastoids. Thus, re-referencing with the mastoids accentuated the noise compared to using the average of all electrodes. Since our participants were not in a Faraday Cage, we performed a spectrum interpolation between the 49 Hz frequency band and the 51 Hz frequency band to remove the 50 Hz noise. To remove artefacts due to eye movements (i.e., saccades and eye blinks), we first ran an Independent Component Analysis (ICA) on 30 components (Mennes et al., 2010). We then selected components corresponding to saccades and eye blinks to be removed based on visual inspection. Finally, we removed the remaining artefacts (i.e., muscular twitch, head movements or flat signal) based on visual inspection. Data were epoched between −0.5 s and 1.2 s around the tone. Then, we used a 300-ms baseline taken between 500 ms and 200 ms before the trigger similar as previous studies (Coll, 2018; Caspar et al., 2022). All event-related potentials were analyzed across Cz and Pz, based on a recent analysis study (Coll, 2018). Based on the same study, only the P3 and the LPP were analyzed as they are more reliably associated with the perception of pain. According to Cheng et al. (2014), we divided the LPP into an early (eLPP) and a late (lLPP) LPP. The P3 was measured as the mean amplitude between the 370 and 470 ms time-window after the tone. The early LPP and the late LPP were measured as the mean amplitude between the 500 and 950 ms time-window and the 950–1,400 ms time-window after the tone, respectively.

## Results

### General statistical analysis

Interval estimates, taken as a proxy for the sense of agency, and ERPs associated with the visualization of the victim’s pain were always analyzed in two ways. In our design, we manipulated the uniform factor across days, with participants wearing a different uniform every day. We thus first realized a repeated-measures ANOVA with Condition (free, coerced) and Clothing (civilian, Red Cross and military) as within-subject factors. The within-subject factor Shock (shock, no shock) was also included for the analyses on time interval estimates, but not for ERPs associated with empathy as they were already computed based on the difference between shock and no shock trials. As we expected a possible reduction of the neural response to the pain of others across days, we also run linear trend analyses across days, irrespective of the uniform wore. Finally, we compared the subjective scores of our participants regarding the three questions asked after each experimental session regarding the outfit they had to wear.

### Prosocial behaviors

**Figure 2:**
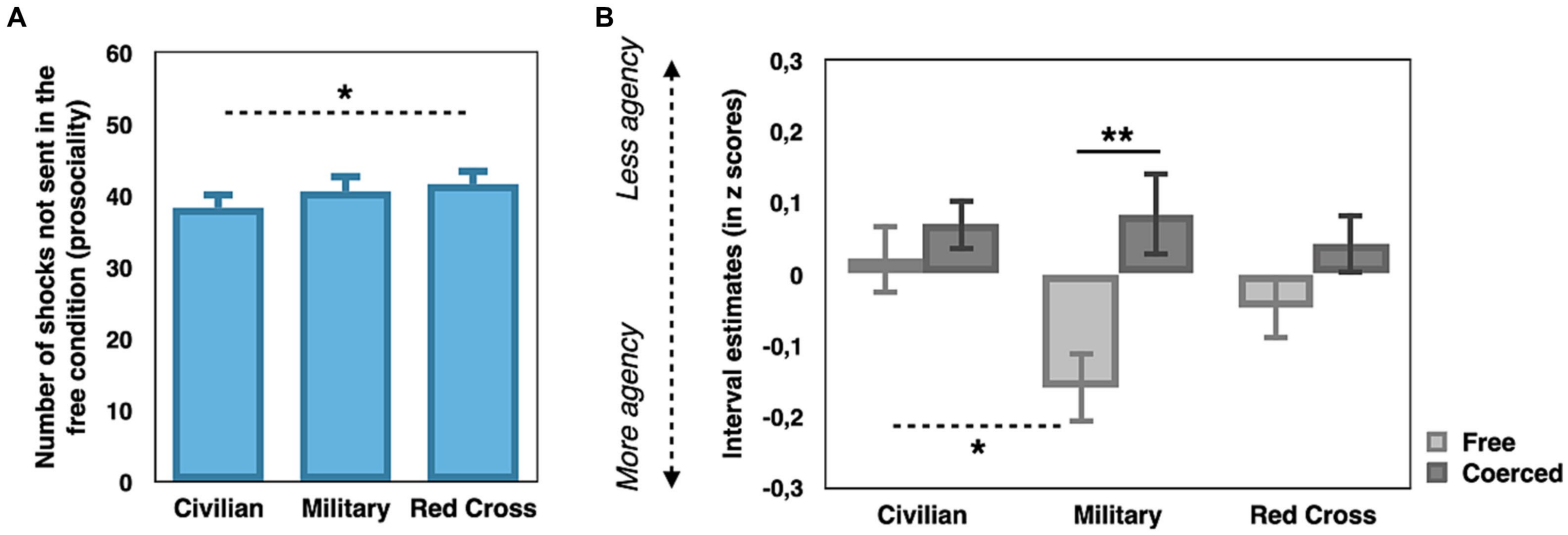
**(A)** Prosocial behavior in the free condition for civilan, Red Cross and military clothings (*N* = 40). **(B)** Z-scored time interval estimates in the free (light grey) and coerced (dark grey) conditions for civilian, Red Cross and military clothing (*n* = 23) dotted lines represent comparisons between different clothing and full lines represent comparisons between experimental conditions. Data are presented as mean value (with error bar = SEM). All test were two-tailed. **Corresponds to a value of *p* between 0.001 and 0.01. *Corresponds to a value of *p* ≤ 0.05.

Prosocial behavior was measured as the number of shocks not sent in the free condition. We first ran a repeated-measures ANOVA with Clothing (civilian, Red Cross, military) as within-subject factor on prosocial behavior. We observed a tendency for a main effect of Clothing (*F*(2,78) = 2.717, *p* = 0.072, *η*^2^_p_ = 0.065). Paired sample *t*-tests were conducted in order to understand if there was a difference in prosocial behavior in the free condition depending on the clothing. We observed that prosocial behaviors were higher when participants wore the Red Cross uniform (*M* = 41.6, *SD* = 15.693) compared to when they wore their civilian outfit (*M* = 38.275, *SD* = 15.693; *t*(39) = 2.428, *p* = 0.020, Cohen’s *d* = 0.384; see Figure 2A). There was no significant difference between the military and Red Cross uniforms, or between the military uniform and the civilian outfit (all *p*s > 0.1). We then ran a repeated-measures ANOVA with days (1, 2, 3) as within-subject factor on prosocial behavior. The main effect of days was not significant (*p* > 0.9). Test of within-subject contrasts revealed no significant linear tendency between days (*p* > 0.7).

### Interval estimates

As participants may have adopted different strategies to report interval estimates over the three testing days, we normalized the data by transforming them into *z*-scores, similarly to previous studies (Caspar et al., 2020b). To note, the LTA, to control for correct discrimination of the three different delays, were done on raw interval estimates prior transformation. We first ran a repeated-measures ANOVA with Condition (free, coerced), Shock (shock, no shock) and Clothing (civilian, Red Cross, military) as within-subject factors on z-scored interval estimates. Consistently with previous studies (Caspar et al., 2016, 2020a), we observed a main effect of Condition (*F*(1,22) = 7.244, *p* = 0.013, *η*^2^_p_ = 0.248), with lower z-scores in the free condition (*z-scor*e = −0.062, *SD* = 0.111) compared to the coerced condition (*z-score* = 0.041, *SD* = 0.111; see Figure 2B). As a reminder, lower *z*-scored interval estimates suggest a higher sense of agency. Neither the main effect of Clothing, nor the main effect of Shock were significant (*p* > 0.4 et *p* > 0.1, respectively). We observed a significant interaction Condition*Clothing (*F*(2,44) = 4.472, *p* = 0.017, *η*^2^_p_ = 0.169). Paired sample t-tests revealed that in the free condition, z-scores were lower when participants wore a military uniform (*z-score* = −0.143, *SD* = 0.246) compared to when they wore the civilian outfit (*z-score* = 0.0297, *SD* = 0.232, *t*(22) = 2.099, *p* = 0.048, Cohen’s *d* = 0.438). Wearing the Red Cross uniform did not significantly differ from wearing the military uniform (*p* > 0.1) or from wearing the civilian outfit (*p* > 0.3). In the coerced condition, none of the paired sample t-tests revealed a statistical difference between the clothing (all *p*s > 0.1). We then ran again paired sample t-tests to evaluate if the ‘coercion effect’ (=free – coerced) differed across the different clothing. We observed that the coercion effect was significant only when participants wore the military uniform (*t*(24) = 2.886, *p* = 0.008, Cohen’s *d* = 0.577), with lower z-scores in the free condition (*z-score =* −0.143, *SD* = 0.246) compared to the coerced condition (*z-score* = 0.015, *SD* = 0.278). None of the other interactions were significant (all *p*s > 0.08). Test of within-subject contrasts revealed no significant linear tendency between Days (*p* > 0.9) or Condition*Days (*p* > 0.6), showing that the observed effects on the interval estimates are stable across days.

### Neural response to the pain of the victim

**Figure 3:**
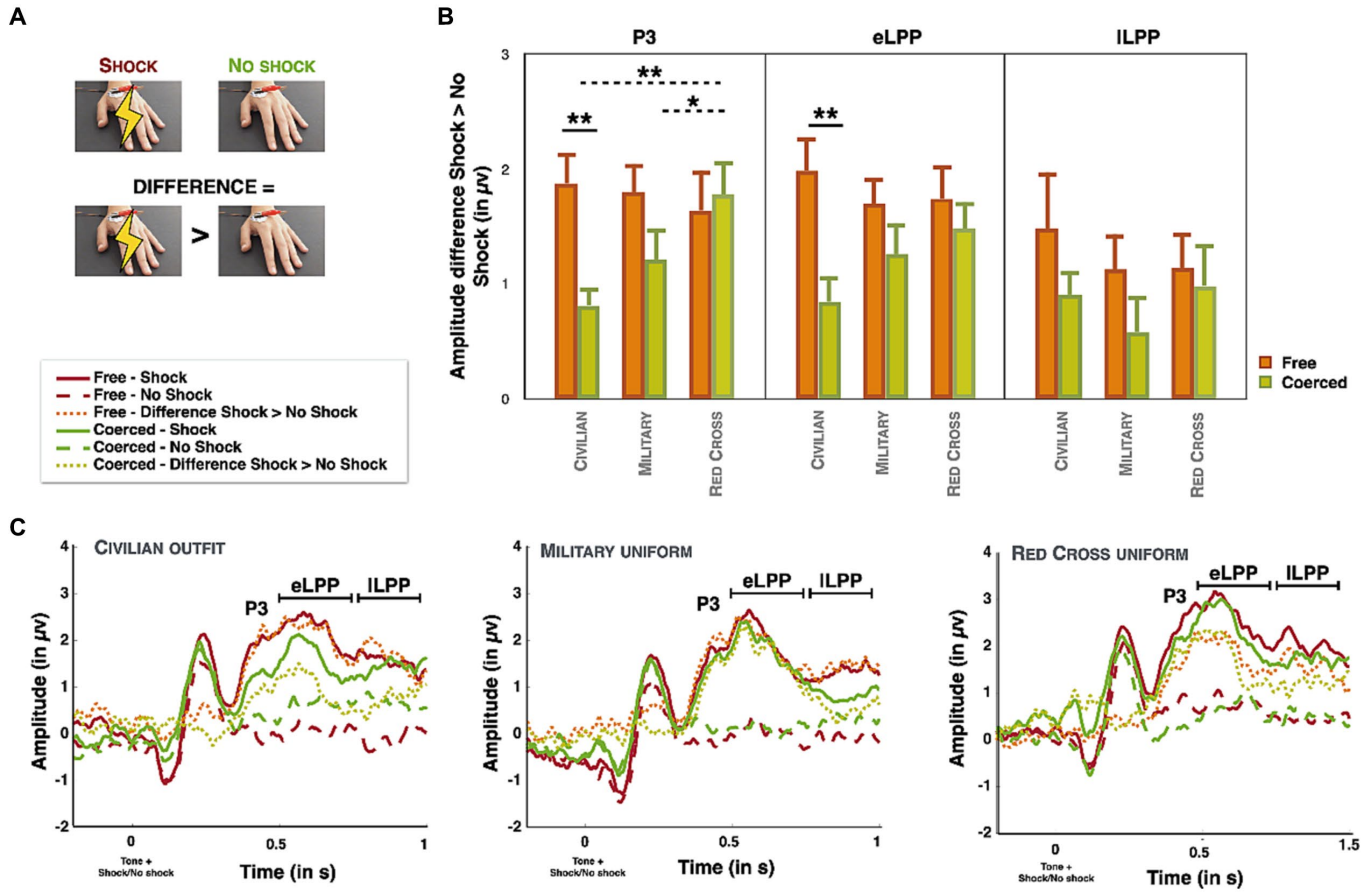
**(A)** Schematic representation of the calculated difference between shock and no shock trials (*n* = 26). **(B)** Graphical representation of the difference of amplitude between shock and no shock trials for the P3, the eLPP and the lLPP for the two experimental conditions (free condition in orange and coerced condition in green) as a mean of the electrodes Cz and Pz. Dotted lines represent comparisons between different clothing and full lines represent comparisons between experimental conditions. Data are presented as mean value (with error bar = SEM). All test were two-tailed. **Corresponds to a value of *p* between 0.001 and 0.01. *Corresponds a value of *p* ≤ 0.05. **(C)** Graphical representation of the Event-Related potentials (P3, eLPP and lLPP) for the three different outfits as a mean of the electrodes Cz and Pz. Full lines represent shock trials, dashed lines represent no shock trials and dotted lines represent the difference.

Separate repeated-measures ANOVA were conducted on the amplitude of the difference between shock and no shocks trials (see Figure 3A) for the P3, the eLPP and the lLPP.

### P3

We observed a main effect of Condition (*F*(1,24) = 4.603, *p = 0*.042, *η*^2^_p_ = 0.161), with a higher amplitude in the free condition(*M* = 1.77 μV, *SD* = 1.10) than in the coerced condition(*M* = 1.27 μV, *SD* = 0.92). There was no significant main effect of Clothing (*p* > 0.3). We observed a significant interaction Condition*Clothing (*F*(2,48) = 4.141, *p = 0*.022, *η*^2^_p_ = 0.147; see Figures 3B,C). Paired sample t-test revealed that in the coerced condition the amplitude of the P3 was higher for the Red Cross clothing (*M* = 1.86 μV, *SD* = 1.52) than for the civilian clothing (*M* = 0.81 μV, *SD* = 0.87, *t*(24) = 2.983, *p* = 0.006, Cohen’s *d* = 0.597) and for the military uniform (*M* = 1.24 μV, *SD* = 1.41, *t*(24) = 2.06, *p* = 0.05, Cohen’s *d* = 0.412). The civilian outfit did not significantly differ from the military clothing (*p* > 0.1). In the free condition there was no significant effect of clothing (all *p*s > 0.6). We then ran again paired sample t-tests to analyse if the ‘coercion effect’ (=free-coerced) differed between the clothing. We observed that the coercion effect was significant only for the civilian clothing (*t*(24) = 3.050, *p =* 0.006, Cohen’s *d* = 0.610), with higher amplitude in the free condition (*M* = 1.92 μV, *SD* = 1.46) compared to the coerced condition (*M* = 0.81 μV, *SD* = 0.87). The coercion effect was not significant for the other uniforms (all *p*s > 0.07). We conducted a repeated-measures ANOVA with Condition and Days in order to investigate the decline of the neural empathic response across days (Decety et al., 2010; Neumann et al., 2011). Test of within-subject contrasts revealed no significant linear tendency across Days (*p* > 0.3) but a significant interaction Condition*Days (*F*(1,24) = 0.689, *p* = 0.025, *η*^2^_p_ = 0.192). We observed that the linear tendency was not significant in the coerced condition (*p* > 0.4) but was significant in the free condition (*F*(1,24) = 5.649, *p* = 0.026, *η*^2^_p_ = 0.191), with a decrease in the amplitude of the P3 across days.

#### eLPP

We observed a main effect of Condition (*F*(1,24) = 7.008, *p =* 0.014, *η*^2^_p_ = 0.226). The amplitude of the eLPP was higher in the free condition (*M* = 1.88 μV, *SD* = 0.88) than in the coerced condition (*M* = 1.23 μV, *SD* = 0.90). No other main effects or interaction effects were significant (all *p*s > 0.1). Test of within-subject contrasts revealed a significant linear tendency across Days (*F*(1,24) = 11.563, *p* = 0.002, *η*^2^_p_ = 0.325) and a significant interaction Condition*Days (*F*(1,24) = 6.085, *p* = 0.021, *η*^2^_p_ = 0.202). Results indicated that the linear trend was significant in the free condition (*F*(1,24) = 20.753, *p* < 0.001, *η*^2^_p_ = 0.464), with a decrease in the amplitude of the eLPP across days. The linear trend was not significant in the coerced condition (*p* > 0.6).

#### lLPP

None of the main effects or their interactions were significant (all *p*s > 0.2). Test of within-subject contrasts revealed no significant linear tendency across Days (*p* > 0.08) and a significant interaction Condition*Days (*F*(1,24) = 10.575, *p* = 0.003, *η*^2^_p_ = 0.306). Results indicated that the linear trend was significant in the free condition (*F*(1,24) = 12.994, *p* = 0.001, *η*^2^_p_ = 0.351), with a decrease in the amplitude of the lLPP across days. The linear trend was not significant in the coerced condition (*p* > 0.2).

### Self-assessment of the effect of clothing

We conducted three separate repeated-measures ANOVA with Clothing (civilian, military, Red Cross) on how participants felt regarding the outfit they wore during each experimental session. For question 1 (i.e., “*How much do I feel that this outfit had an effect on my behaviors?*”), we observed a main effect of Clothing (*F*(2,78) = 7.922, *p* = 0.001, *η*^2^_p_ = 0.169). Paired-sample t-tests revealed that participants reported a higher effect on their behaviors with the military uniform (*M* = 1.9, *SD* = 2.01, *t*(39) = 3.991, *p* < 0.001, Cohen’s *d* = 0.631) and the Red Cross uniform (*M* = 1.525, *SD* = 2, *t*(39) = 3.129, *p* = 0.003, Cohen’s *d* = 0.495) compared to their civilian outfit (*M* = 0.67, *SD* = 1.47). There was no difference between the military and the Red Cross uniforms (*p* > 0.3). For question 2, (i.e., “*How much do I feel that my state of mind was influenced by this outfit?*”), we observed a main effect of Clothing (*F*(2,78) = 4.429, *p* = 0.015, *η*^2^_p_ = 0.102). Paired-sample t-tests revealed that participants reported a higher change of their state-of-mind with the military uniform (*M* = 2.07, *SD* = 1.99, *t*(39) = 3.150, *p* = 0.003, Cohen’s *d* = 0.498) and the Red Cross uniform (*M* = 1.850, *SD* = 1.99, *t*(39) = 2.100, *p* = 0.042, Cohen’s *d* = 0.332) compared to their civilian outfit (*M* = 1.12, *SD* = 1.76). There was no difference between the military and the Red Cross uniforms (*p* > 0.5). For question 3, (i.e., *“To what extent I behave as a military/first aid worker/civilian during the experimental session?”*), we observed a main effect of Clothing (*F*(2,78) = 17.684, *p* < 0.001, *η*^2^_p_ = 0.312). Paired-sample t-tests revealed that participants reported behaving less in accordance with their outfit with the military uniform (*M* = 2.9, *SD* = 1.92, *t*(39) = 3.501, *p* = 0.001, Cohen’s *d* = 0.554) and the Red Cross uniform (*M* = 2.32, *SD* = 1.71, *t*(39) = 6.607, *p* < 0.001, Cohen’s *d* = 1.045) compared to their civilian outfit (*M* = 4.425, *SD* = 2.11). There was no difference between the military and the Red Cross uniforms (*p* > 0.08).

### Exploratory correlation between self-assessment and other measurements

We conducted exploratory Pearson correlations between the three questions and the other measurements. First, we investigated the consistency of the questions among them to possibly create a unified item. We calculated Cronbach’s alpha for each unit separately by considering all combinations of the questions: Q1 with Q2 and Q3, Q2 with Q3, and Q1 with Q3. We retained the combination that exhibited higher consistency and was above 0.7, indicating reliability (Gefen et al., 2000). For all clothing types, Cronbach’s alpha revealed that combining Q1 and Q2 resulted in the highest score (civilian: α = 0.822; all other combinations αs < 0.61; Red Cross: α = 0.855; all other combinations αs < 0.854; military: α = 0.960; all other combinations αs < 0.762). Consequently, we grouped Q1 and Q2 for each uniform to represent the perceived effect produced by wearing the uniform (referred to as Qeffect), while Q3 remained separate for the perception of playing a role associated with the uniform (referred to as Qrole). Subsequently, we conducted Pearson correlations with Qeffect and Qrole separately on the number of shocks prevented, interval estimates (for shock and no shock), P3, eLPP, and lLPP for each uniform. After adjusting for multiple comparisons using the False Discovery Rate (FDR, Benjamini and Hochberg, 1995), none of the correlations were found to be statistically significant (all *p*FDRs > 0.07).

## Discussion

Does the uniform we wear influence our behavior? Adam and Galinsky (2012) addressed this question through a novel theoretical framework known as enclothed cognition. The purpose of enclothed cognition is to explore how the symbolic meaning of clothes impacts our internal psychological processes and behavioral tendencies. According to this theory, wearing symbolic clothes activates abstract concepts and their associated meanings, subsequently influencing our cognition and behavior. To contribute to this body of research, we examined the effects of wearing symbolic uniforms on prosocial behavior, as well as two related neuro-cognitive processes: the sense of agency and empathy for pain. Volunteer participants wore their own civilian outfit, a Red Cross uniform, and a military uniform on three consecutive days. In the role of an agent, they had the choice to freely decide or were coerced to administer real but mildly painful electric shocks to another participant in exchange for a small monetary compensation. Our findings revealed that wearing the Red Cross uniform resulted in increased prosocial behavior, as indicated by a higher number of shocks prevented from administration to the other participant in the free decision condition, compared to wearing a civilian outfit. Additionally, wearing the Red Cross uniform enhanced the neural empathic response under coercion, in contrast to wearing the military uniform or a civilian outfit. When participants wore the military uniform, we observed a heightened sense of agency when agents were allowed to freely decide their actions, as opposed to wearing a civilian outfit. Finally, we also noted a decline in the neural empathic response to the pain experienced by the victim across the three testing days.

Prosocial behaviors were measured by the frequency of participants preventing the administration of a painful shock to the other participant by refusing the small monetary gain. Previous studies have shown an increase in prosociality when individuals wore healthcare uniforms compared to other types of uniforms (Johnson and Downing, 1979; López-Pérez et al., 2016). Therefore, we anticipated that wearing the Red Cross uniform, representing healthcare, would enhance prosociality compared to the other uniforms (i.e., military uniform and civilian outfit). Our results confirmed this hypothesis, demonstrating that wearing a Red Cross uniform led to a greater level of prosociality compared to wearing their own civilian outfit. This finding both replicates the work of Johnson and Downing (1979) and extends the findings of López-Pérez et al. (2016) regarding the impact of uniforms on prosocial behaviors. One possible explanation for this effect is that healthcare uniforms are associated with qualities such as caring, kindness, or sympathy (Petrilli et al., 2015; Tiang et al., 2017), which could enhance prosocial tendencies. Interestingly, wearing the military uniform did not appear to influence prosociality, as there was no significant difference in the number of shocks withheld between wearing the military uniform and the civilian outfit. This lack of effect suggests that the military uniform may not be perceived in the same manner as the Red Cross uniform. This notion is supported by the findings of Niederhauser et al. (2009), who observed that military patients reported greater comfort and were more likely to return to medical offices when their military doctor wore a healthcare uniform instead of a military uniform. These results indicate that military uniforms may be less associated with caring and prosocial behaviors compared to healthcare uniforms. However, due to the limited existing literature on this topic, this is merely an assumption, and future studies on enclothed cognition should further explore how individuals perceive the symbolic meaning of the uniforms they wear and how it relates to their decisions to act prosocially or not.

Previous literature has consistently indicated that empathy is a crucial neuro-cognitive process associated with prosocial behaviors (Hein and Singer, 2010; Davis, 2015). In this study, we sought to investigate how wearing different symbolic uniforms influenced the neural empathic response to others’ pain. Building upon previous research (Cheng et al., 2014) and a recent meta-analysis (Coll, 2018), we focused on three Event-Related Potentials (ERPs) - the P3, eLPP, and lLPP - known for their sensitivity to the experience of others’ pain. Recent findings (Caspar et al., 2022) have also observed that these ERPs are generated in the insula and anterior cingulate cortex, similar to the activation patterns observed in MRI studies on empathy for pain (Timmers et al., 2018; Jauniaux et al., 2019), further supporting their association with the neural response to others’ pain. To note, in this study the ERPs are the subtraction of the no shock trial to the shock trial. This subtraction was made in order to focus on the empathic component of the ERPs as they could also reflect process that are both present in the shock and no shock trials. In our study, we initially observed a reduction in the amplitude of the P3 and eLPP when participants were coercively instructed compared to when they had the freedom to choose. These results confirm that obeying orders diminishes our empathic capacity for the pain we inflict on others compared to acting freely (Caspar et al., 2020a). Furthermore, we found that wearing the Red Cross uniform led to an increased amplitude of the P3 in the Coerced condition, in contrast to wearing their civilian outfit or the military uniform. López-Pérez et al. (2016) previously reported that wearing a healthcare uniform was associated with higher self-reported empathy compared to wearing a cleaner uniform. Our results extend their findings by demonstrating that the increase in empathy observed when participants wore a healthcare uniform can also be observed at the neural level. Interestingly, although military uniforms are often perceived as symbols of protection and care for others (Taylor et al., 2011), we did not find a significant difference in the neural empathic response between wearing the military uniform and the civilian outfit. This suggests that the symbolic meaning of the military uniform may not have been potent enough to elicit an increase in the neural empathic response. Collectively, our results indicate that the extent to which uniforms are associated with caring and prosocial concepts influences their impact on the neural response to others’ pain. This increase in the neural empathic response when wearing the Red Cross uniform could also account for the observed increase in prosociality with the same uniform, as empathy for pain is considered a key neuro-cognitive process underlying prosocial behaviors (Hein and Singer, 2010; Gallo et al., 2018).

Importantly, the influence of wearing uniforms on the ERPs associated with the experience of others’ pain was evident in the coerced condition but not in the free condition. One possible explanation is that the amplitude of these ERPs in the free condition was influenced by the three consecutive testing days. Trend analysis revealed a significant decrease in the amplitude of the P3, eLPP, and lLPP across the 3 days of testing in the free condition. These findings align with previous studies demonstrating a decline in the empathic response to others’ pain among physicians or medical students over time (Decety et al., 2010; Neumann et al., 2011; Smith et al., 2017). One possible explanation for this phenomenon is the necessity for healthcare professionals to regulate their empathy in order to protect themselves from personal distress and compassion fatigue arising from witnessing the pain of others (Decety et al., 2010). The ability to regulate empathy and attenuate it when necessary allows healthcare professionals to utilize other cognitive resources that are essential for providing care and healing to patients. Another explanation could be attributed to a habituation effect. Numerous studies have shown that repeated exposure to violence can lead to a reduction in empathy (Guo et al., 2013; Tarabah et al., 2016). In our study, the visual presentation of painful shocks over three consecutive days might have induced a habituation phenomenon, resulting in a decrease in the neural empathic response over time. Regarding our results, the potential impact of clothing in the free condition may have been attenuated due to the confounding factor of the testing day. To address this limitation, one experimental possibility would involve introducing longer intervals between consecutive testing days to minimize the habituation effect.

Consistent with previous studies, we found that the sense of agency, measured using interval estimates as an implicit method, was diminished in the coerced condition compared to the free condition (Caspar et al., 2016, 2020b). In a previous study by Caspar et al. (2020b), it was observed that military privates and junior cadets exhibited reduced sense of agency in the free condition compared to civilians or senior cadets (i.e., officers). However, in that study, military participants wore their military uniform during testing while civilians wore their own civilian outfit. Consequently, it is possible that the observed effects were attributed to clothing rather than the military environment itself. In the present study, we found that the sense of agency was higher when participants wore a military uniform in the free condition compared to when they wore their own civilian outfit. This finding not only supports the notion that the effects observed (Caspar et al., 2020b) were indeed linked to the military environment and training rather than clothing. A plausible explanation for the heightened sense of agency while donning a military uniform is the association of these uniforms with the concept of responsibility. Notably, a previous survey reported that 78% of Americans expressed confidence in the military, ranking it highest among 16 institutions, indicating that the military institution is often associated with a high level of responsibility (Jones and Saad, 2011). This survey suggests that the military institution is commonly linked to a heightened sense of responsibility. However, there are a couple of important considerations. First, the survey was conducted exclusively among Americans, potentially rendering its findings non-representative of perceptions within Belgium. Secondly, we acknowledge that that this proposition is an assumption, as there exists limited extant literature delving into the interplay between uniform-associated concepts and the resultant cognitive effects. Furthermore, our study did not explicitly inquire about the concepts evoked by the participants in response to the uniforms. Hence, we recommend that future investigations into enclothed cognition include this query. Such an approach would contribute to a better understanding of the specific concepts elicited by uniforms and their interplay with the cognitive effects stemming from wearing them.

Interestingly, participants reported a subjective perception that wearing the military uniform and the Red Cross uniform had a greater influence on their behavior and state of mind compared to wearing their civilian outfit. However, the average scores for these two questions, rated on a scale from 0 (not at all) to 6 (very much), were below 3. This suggests that participants tended to disagree with the notion that uniforms significantly altered their behavior and state of mind. Nonetheless, our results did indicate that wearing symbolic uniforms did influence prosocial behaviors and cognition. It is possible that participants may not be fully aware of the impact of wearing symbolic uniforms on their behavior and cognition. Additionally, it is plausible that certain individuals may be more sensitive to the symbolic meaning of their clothing than others. In the third question, we specifically asked participants to what extent they acted in accordance with the outfit they wore on each day. This inquiry aimed to assess whether our findings were a result of participants consciously modifying their behaviors and state of mind to align with the roles associated with military and first aid workers. Results indicated that participants generally agreed that when they wore their civilian outfit, they behaved as civilians. However, for the military uniform and the Red Cross uniform, participants mostly disagreed that they acted as military personnel or first aid workers. This finding suggests that our results are not solely attributable to demand characteristics (Orne, 2009) but instead reflect genuine effects of wearing specific uniforms on behaviors and cognition.

However, this study faced some limitations that should be acknowledged. Firstly, participants may have become aware of the purpose of the study after the first day of testing. By asking participants to estimate the effects of the uniforms from the end of the first test day, they may have inferred the study’s objective and attempted to please the experimenter by conforming to the expected behavior associated with their clothing in the next days. However, it is important to note that we randomized the uniforms across days among participants, mitigating potential bias. Furthermore, participants generally disagreed with the notion that the uniform could have influenced their behavior and disagreed when asked if they acted in accordance with the uniforms worn, suggesting that demand characteristics were unlikely to have significantly impacted our results. A second limitation is that we did not inquire about participants’ prior experience of wearing these uniforms, which could have influenced the outcomes. However, we specifically recruited individuals who were not military or first aid Red Cross workers, as outlined in the inclusion criteria. Nevertheless, future studies should consider controlling the potential effect of prior exposure to symbolic clothing. A third potential limitation could be the possibility that the observed effect is influenced by the victim’s uniform, instead of the agent’s uniform. Both the agent and the victim wore identical uniforms when they dressed at the beginning of the experiment. However, it is highly unlikely that our results are attributed to the victim’s uniform, as the agent was not physically present in the same room as the victim during the task. Furthermore, the agent could only observe the victim’s hand through the camera, where the uniform was not discernible (see Figure 1). Nevertheless, future studies may address this potential effect by having the agent dressed differently from the victim to control for this variable. A fourth limitation could be that the observed effects result from changes in the experimenter’s attitude due to expectations of the effect (Doyen et al., 2012). To prevent such influence in our data, we provided the exact same instructions regardless of the uniform participants wore, and we mainly relied on implicit measurements. Nevertheless, we recommend that future studies investigate the possibility of experimenter expectation influence. A control for this possibility is the implementation of double-blind experiments. Finally, although we recruited 40 participants for the present study, three more than the estimated sample size to account for potential data loss, the exclusion rate was higher than expected. Thirteen participants did not send enough shocks on at least one testing day, resulting in their exclusion from the entire experimental procedure due to the absence of data in the free condition. The number of data excluded in the present study is mostly due to the within-subject design, as not sending enough shocks on 1 day prevented to use their full data set in the analyses. The low number of shocks that participants decided to inflict, leading to data loss, may be related to our three consecutive day-experimental design. As they were seeing the same co-participant over 3 days, this may have increased their bonding, perspective-taking and feeling of ingroup, which are known to decrease antisocial conducts (Galinsky and Moskowitz, 2000; Montoya et al., 2008; Lemay and Ryan, 2021). Future studies could consider mixing pairs of participants. Additionally, four of the remaining participants did not exhibit a significant trend across the three intervals used, leading to their exclusion from the interval estimate analysis. As a result, some of our effects may have been underpowered.

Despite these limitations, the present study expands upon previous research on the influence of wearing symbolic uniforms on cognition and behaviors, revealing their effects on prosocial behaviors, empathy for pain, and the sense of agency. Our findings indicate that different uniforms have varying effects on agency and empathy for pain, supporting the notion that it is the specific symbolism of the uniforms rather than the mere act of wearing uniforms that influences cognition. Importantly, some argue that there is a wealth of evidence that challenges the embodied cognition framework (Goldinger et al., 2016; Zwaan, 2021). It is crucial to recognize that asserting support for or against the embodied cognition framework requires caution, given its status as a non-falsifiable ‘research program,’ much like connectivism or cybernetics (Lakatos, 1976; Wołoszyn and Hohol, 2017). Research programs are designed to develop falsifiable theories; however, research programs themselves cannot be falsified. However, the enclothed cognition theory, which posits that the symbolic meaning of clothing can modify psychological processes, is falsifiable and our results provide support for this theory (Adam and Galinsky, 2012).

## Data availability statement

The datasets presented in this study can be found in online repositories. The names of the repository/repositories and accession number(s) can be found at: https://osf.io/5st2m/.

## Ethics statement

The studies involving humans were approved by the Ethics Advisory Committee of the Faculty of Psychology and Education ULB-Erasme. The studies were conducted in accordance with the local legislation and institutional requirements. The participants provided their written informed consent to participate in this study. Written informed consent was obtained from the individual(s) for the publication of any identifiable images or data included in this article.

## Author contributions

GP: Conceptualization, Data curation, Formal analysis, Investigation, Methodology, Software, Visualization, Writing – original draft. EC: Conceptualization, Funding acquisition, Project administration, Resources, Supervision, Writing – review & editing.
